# Bisphenol A and Male Fertility: Myths and Realities

**DOI:** 10.3389/fendo.2020.00353

**Published:** 2020-06-12

**Authors:** Chiara Castellini, Maria Totaro, Antonio Parisi, Settimio D'Andrea, Liana Lucente, Giuliana Cordeschi, Sandro Francavilla, Felice Francavilla, Arcangelo Barbonetti

**Affiliations:** Medical Andrology, Department of Life, Health and Environmental Sciences, University of L'Aquila, L'Aquila, Italy

**Keywords:** endocrine disruptors, environmental pollution, oxidative stress, spermatozoa, sperm DNA damage

## Abstract

Bisphenol A (BPA) represents the main chemical monomer of epoxy resins and polycarbonate plastics. The environmental presence of BPA is widespread, and it can easily be absorbed by the human body through dietary and transdermal routes, so that more than 90% of the population in western countries display detectable BPA levels in the urine. As BPA is qualified as an endocrine disruptor, growing concern is rising for possible harmful effects on human health. This review critically discusses the available literature dealing with the possible impact of BPA on male fertility. In rodent models, the *in vivo* exposure to BPA negatively interfered with the regulation of spermatogenesis throughout the hypothalamic–pituitary–gonadal axis. Furthermore, in *in vitro* studies, BPA promoted mitochondrial dysfunction and oxidative/apoptotic damages in spermatozoa from different species, including humans. To date, the claimed clinical adverse effects on male fertility are largely based on the results from studies assessing the relationship between urinary BPA concentration and conventional semen parameters. These studies, however, produced controversial evidence due to heterogeneity in the extent of BPA exposure, type of population, and enrollment setting. Moreover, the cause–effect relationship cannot be established due to the cross-sectional design of the studies as well as the large spontaneous between- and within-subject variability of semen parameters. The best evidence of an adverse effect of BPA on male fertility would be provided by prospective studies on clinically relevant endpoints, including natural or medically assisted pregnancies among men either with different exposure degrees (occupational/environmental) or with different clinical conditions (fertile/subfertile).

## Introduction

Bisphenol A (BPA), 4,40-isopropylidenodi-phenol, 2,2-bis(4-hydroxyphenylo)propane, is a crystalline chemical compound widely used as key monomer of epoxy resins and polycarbonate (PC) plastics for more than 50 years (1). The industrial use of BPA is impressive with ~9 million tons per year produced worldwide (2–4). Resiliency, flexibility, and durability have decreed the large-scale success of BPA-based PC plastics, leading to their use in many and various fields, ranging from the arms industry, for components of safety equipment (helmets), to the manufacture of medical devices, including dental sealants and fillers. In the food industry, synthetic materials containing BPA are widely employed for manufacturing long-term food and drink containers and represent key components of protective coatings, including those covering the internal surface of cans (2–4). A wide variety of other commonly used articles also contain BPA and its derivatives: fridges, baby bottles, dishes, lenses, sunglasses, hair dryers, CD and DVD, cell phones, computers, and thermal paper (1, 5, 6). Owing to its ubiquitous presence, environmental persistence, and the reputation of being an endocrine disruptor, BPA is now regarded as a potential threat to human health, and concerns arise from its possible link with cardiovascular diseases, metabolic disorders, cancer, and infertility (7–10).

The aim of this review is to critically outline and discuss the available literature dealing with the possible impact of BPA on male fertility.

## Toxicokinetics of BPA

BPA is an ideal plasticizer because of its cross-linking characteristics; nevertheless, free monomers can be released into food content after polymerization, especially on exposure to high temperature and with re-use of the containers: this makes possible BPA entering the organism (11–14). As early as 1994, Brotons et al. (15) reported that vegetables preserved in lacquer-coated cans acquire estrogenic activity due to contamination by BPA, which is leached from the lacquer coating. More recently, Kubwabo et al. (16) reported that BPA also migrated from the wall of PC baby bottles: high temperatures and prolonged incubation times resulted in increased leaching of BPA, especially when fatty foods were used, whereas BPA leaching from non-PC baby bottles appeared to be negligible under the same experimental conditions (16). Hence, as a precautionary measure, the European Union banned BPA in the production of baby bottles in 2011(2011/8/EU). Resin-based dental filling materials have been feared as another possible source of oral exposure (17). According with two recent systematic reviews, BPA can leach from some resin-based dental materials into the saliva (18, 19), reaching detectable urinary concentrations that peak 24 h after treatment (19). However, the extent to which such an increase may affect the health of patients remains an open question (18).

According to the main regulatory agencies, dietary route represents the primary source of human exposures (2, 20–26) and a tolerable daily intake (TDI) of 50 μg/kg body weight/day has been established, based on studies from rodent models, where clear harmful effects at much higher doses were registered. Based on an analysis of consumer exposure to BPA, the European Food Safety Authority (EFSA) stated that the current levels of exposure are below the TDI (27). Therefore, they would not represent a threat for consumers at any age, taking also into account the short half-life of orally ingested BPA (27).

After ingestion, most of BPA is quickly bound to glucuronic acid by the liver enzyme uridine diphosphonate glucuronosyl transferase (UGT) to produce BPA glucuronide (BPA-G) (25, 28). This rapid first-pass liver metabolism makes BPA more soluble in water, with a half-life of elimination in urine of 5.4–6.4 h (28, 29). Therefore, at oral doses ranging from 50 to 100 μg/kg body weight (far above the TDI), in humans, BPA elimination is essentially complete within 24 h, with free BPA accounting for <1% of total PBA (28, 29). Of note, toxicokinetic processes can be influenced by physiological changes related to pregnancy, as the placenta exhibits beta-glucuronidase enzymatic activity that deconjugates BPA-G (26, 30, 31). Once having crossed the placental barrier, the BPA conversion to BPA-G in the fetus would be poorly effective, due to the immature liver functions (30).

The highly effective detoxifying system of the human body could counteract possible consequences of a large-scale exposure to BPA. In line with the ubiquitous use of this substance, the reports by the Center for Disease Control and Prevention revealed that more than 90% of the US population displays detectable BPA levels in urine (32, 33). In the largest population study by Calafat et al. (34), detectable levels of total (free plus conjugated) BPA were found in 92.6% of urine samples from 2,517 participants aged ≥6 years in the 2003–2004 National Health and Nutrition Examination Survey (NHANES). According to a recent study by Gerona et al. (35), these epidemiological data could even be underestimated. Indeed, the results of the largest population studies were produced by using indirect methods requiring the enzymatic hydrolysis of conjugated BPA to free BPA, before its quantification in urine by liquid chromatography–mass spectrometry (LC-MS). Unfortunately, the deconjugation process would be largely incomplete in many cases (35).

Interestingly, when NHANES data were adjusted for the fasting hours preceding the collection of the urinary samples, no clear inverse relationship between fasting hours and urinary BPA levels was found (36). This finding seems to be at the variance with the assumption that BPA is rapidly eliminated after ingestion and that the digestive tract represents the main source of exposure. On the contrary, NHANES data could suggest that either the half-life of BPA is longer than we think or this substance can, to some extent, remain stored in the body or it can even be assimilated through alternative non-dietary routes. Indeed, BPA is also detectable in indoor/outdoor air and floor dust and is widely used in products that come into contact with skin, including not only cosmetics but also thermal paper, where BPA is used as a heat-activated developer (2, 27, 37). This makes possible its absorption by alternative non-dietary means, such as inhalation and transdermal route. While the estimated inhalation exposure would be negligible when compared to dietary route (38, 39), with the exception of some factory workers with high occupational exposure (40, 41), transdermal absorption should deserve special attention. Unlike plastics and can linings, where BPA is largely in a polymerized form (PC or PVC), the printing surface of many thermal papers contains milligrams of unbound (free) BPA per gram of paper (42–45), thus explaining the quick transdermal absorption of BPA from this source after handling (26, 45–47): this raises special concerns in individuals who work as cashiers (48). The absorption degree is further enhanced by chemicals which are present in hand sanitizers that can cause a breakdown of the dermal barrier (45). According to EFSA (49), apart from oral exposure, the skin contact with thermal paper represents a major source of exposure to BPA. Of note, while almost all of bloodstream circulating BPA following oral ingestion is in the conjugated form (50–52), after entering the body via a transdermal route, BPA bypasses the liver metabolism, resulting in significantly higher concentrations of unconjugated form in the bloodstream (50, 53–56). This is relevant to toxicodynamics because only unconjugated BPA can activate estrogen receptors and is regarded as the biologically active one.

## Effects of BPA on Male Fertility

### Effects on Spermatogenetic Function: Evidence From Preclinical Studies

In the last decades, results of preclinical research revealed endocrine-disrupting effects of BPA on male reproductive functions, clarifying possible mechanisms by which BPA can interfere with the regulation of spermatogenesis mainly throughout the hypothalamic–pituitary–gonadal axis.

In rodent models, with some exceptions (57–59), the *in vivo* exposure to BPA at different doses (largely ranging from 2 μg/kg/day to 960 mg BPA/kg body weight/day) and time intervals (from 5 to 84 days) resulted in a significant decrease in sperm counts (60–70), sperm motility (61, 62, 67), normal sperm morphology (62), increase in sperm DNA damage (63, 67), and poor spermatogenesis (64–66, 70–72). A large between-studies heterogeneity in both cumulative effective doses and tolerable daily intakes was observed. It might partially be due to differences in susceptibility to BPA effects across rodent species and strains. Genetic factors of animal models can modulate the metabolic rate of a chemical substance, accounting for the variability of its toxicokinetics among the species (73, 74). This could determine a variable extent of sensitivity of different species and strains to the same chemical under the same experimental conditions (75).

An interference at hypothalamic–pituitary level of the gonadal axis has been clearly demonstrated in the rat, where, with a few exceptions (65, 76), the administration of BPA significantly lowered both the expression of the GnRH gene in cells of preoptic area (64) and circulating levels of gonadotropins and/or testosterone (64, 69, 70, 77–81). Interestingly, the perinatal phase would represent a sensitive exposure window (3), as the treatment of pregnant and nursing dams with BPA decreased intratesticular (77) and circulating (82) testosterone levels of male offspring in adulthood.

BPA is qualified as a xenoestrogen because it mimics estrogen effects due to its characteristic polycyclic phenolic chemical structure, similar to estradiol (77). In a study by Matthews et al. (83), BPA, but not the soluble product of its glucuronidation, was able to displace tritiated 17-β estradiol from the estrogenic alpha and beta receptors (ERα and ERβ, respectively). The authors observed a more evident dose dependence for ERβ, to which BPA exhibited higher affinity than to ERα (83). The affinity for ERα is 10,000 times lower than that of 17-β estradiol, more than 20,000 times lower than that of diethylstilbestrol, a synthetic molecule with a powerful estrogenic activity, and 3–700 times lower than that of various polychlorinated biphenyls, which represent ubiquitous organic polluting compounds in the environment (84). Despite the low affinity, the binding of BPA to ERs is biologically functional in terms of ER-dependent transcription of target genes, as demonstrated by the luciferase reporter gene assay (83). Noteworthy, although BPA acts as a weak estrogen on ERs, it exhibits a very higher affinity (similar to estradiol) for the membrane G protein-coupled estrogen receptor (GPER) of the non-classical estrogenic pathways, mediating rapid non-genomic effects of BPA even at low doses (85). In males, such an estrogen-like endocrine disruption is expected to interfere with the feedback mechanisms of the hypothalamic–pituitary–gonadal axis, leading to a reduced pituitary secretion of gonadotropins and consequent hypostimulation of spermatogenesis and Leydig cell steroidogenesis.

Indeed, the decrease in testosterone levels in animals exposed to BPA could reflect a combination of central (hypothalamic–pituitary) and peripheral (testicular) effects. The *in vitro* treatment of Leydig cells from adult rat with BPA decreased testosterone biosynthesis as a result of decreased expression of steroidogenic enzymes (77, 86).

Further possible mechanisms leading to an androgen deficiency status could be sought in the endocrine perturbation exerted by BPA on the differentiation and functions of the adipose tissue. BPA promotes both adipogenesis (87) and lipid storage in adipocytes (88); furthermore, animals treated with low doses of BPA exhibited obesity-related metabolic dysfunctions (89). In this view, BPA is now regarded as a possible environmental obesogen (90). In the complex and bidirectional relationship between obesity and low testosterone, it is well-demonstrated that adipocytes express aromatase activity which is responsible for testosterone conversion into estradiol (91), which can exert a synergistic inhibitory effect on pituitary secretion of luteinizing hormone (LH) (92, 93). An excess of fat mass is also associated with increased levels of circulating leptin which exerts a direct inhibition of Leydig cell steroidogenesis (94, 95). Noteworthily, the chemical structure of BPA is lipophilic; therefore, the effects on adipocytes could be amplified and maintained by its retention in fat mass, establishing a possible vicious circle (96).

BPA can also exert anti-androgenic activity by interfering with the signaling of the androgen receptor (AR) at several levels (9, 69, 97). BPA acts as a competitive (98) and non-competitive (99) antagonist of AR and decreases the expression of AR in the testis (66). Other mechanisms of the anti-androgenic interference include the disruption of the nuclear AR translocation (99) and the enhancement of the interaction of AR with its corepressors, such as the silencing mediator of retinoid and thyroid hormone receptor (SMRT) and the nuclear receptor co-repressor (N-CoR) (100). As spermatogenesis requires both high intratesticular levels of testosterone and an adequate functionality of the AR (101, 102), it is not surprising that the effects of BPA on testosterone biosynthesis and activity could affect spermatogenic function.

Independently of its hormonal disrupting effects, BPA could interfere with spermatogenesis processes even through other mechanisms. After *in vivo* exposure to BPA, an impaired testicular glucose homeostasis has been reported in the rat (103), and an increased testicular oxidative stress has been revealed both in the rat (70, 103) and in the mouse (104, 105). BPA can also induce apoptosis in cultured Sertoli cells from rodents (106–109) by inducing dysfunction of mitochondria and generation of reactive oxygen species (ROS) (110). Moreover, an impaired expression of junctional proteins of Sertoli cell has been found in rats that were exposed to BPA neonatally (61), while a downregulated expression of genes involved in Sertoli cell functions has been found in mice that were exposed to BPA prenatally (62).

### Effects on Semen Quality and Reproductive Outcomes: Evidence From Clinical Studies

Due to the obvious lack of controlled clinical trials investigating the effects of BPA on human male fertility, information is largely inferred from findings of observational epidemiological studies that, with a few exceptions, used semen quality as a surrogate endpoint, producing divergent results likely due to heterogeneity in the extent of BPA exposure, sample sizes, type of population, and enrollment setting (Table 1). Some studies included men from the general population (112–115), others included men attending fertility clinics with (116–120) or without known subfertility (121); one study was restricted to men with proven fertility (122). Only in two studies were men with occupational exposure to BPA included (111, 112).

**Table 1:** Epidemiological studies on the relationship of urinary BPA concentration with semen quality and/or other reproductive outcomes.

**Study**	**Population**	**BPA urinary concentration: mean ± SD or median (range)**	**Results**	**Adjustments**
Mendiola et al. (107)	Fertile men (*n =* 375)	1.5 (0.80–3.0) μg/l^*^	No significant associations between urinary BPA and semen parameters.	Age, BMI, smoking status, ethnicity, center, urinary creatinine concentration, and time to motility analysis.
Meeker et al. (101)	Male partners of subfertile couples (*n =* 190)	1.3 (1.8–2.5) ng/ml	Urinary BPA concentrations were linearly associated with lower percentages of sperm with normal morphology (β regression coefficient: −0.90, 95% CI: −1.79, −0.004, *p =* 0.049), lower VCL values (β regression coefficient: −3.97, 95% CI: −7.65, −0.27, *p =* 0.04) and increased DNA damage (β regression coefficient: 3.88, 95% CI: 0.01, 7.74, *p =* 0.048).	Age, BMI, abstinence period, and smoking.
Li et al. (97)	Factory workers with and without occupational BPA exposure (*n =* 218)	38.7 (6.3–354.3) μg/grCr in exposed and 1.4 (0.0–17.9) μg/grCr in non-exposed	Urinary BPA was associated with lower sperm concentration (β regression coefficient: −15.6; *p* < 0.001), total sperm count (β regression coefficient: −42.1; *p =* 0.004), sperm vitality (β regression coefficient: −4.6; *p* < 0.001), and motility (β regression coefficient: −3.1; *p* < 0.001).	Age, education, history of chronic disease, previous exposure to other chemicals and heavy metals, employment history, marital status, age at first intercourse, smoking, alcohol consumption, and center.
Chen et al. (105)	Infertile men (*n =* 877) and fertile controls (*n =* 713)	Geometric means: 0.612 ng/ml in cases and 0.621 ng/ml in controls	No significant associations between urinary BPA levels and standard semen parameters	Age, BMI, and urinary creatinine concentration.
Buck Luis et al. (109)	Couples recruited upon discontinuing contraception to become pregnant (*n =* 501)	1.04 (0.91–1.18) ng/ml^*^	BPA concentration was not associated with time to pregnancy.	Partner age, BMI and urinary creatinine concentration, female urinary BPA concentration, smoking, and center.
Knez et al. (102)	Male partners of couples seeking infertility treatment (*n =* 149)	1.55 (0.81–3.27) ng/ml^*^	Urinary BPA was associated with lower total sperm count (β regression coefficient: −0.241, 95% CI: −0.47, −0.012), sperm concentration (β regression coefficient: −0.219, 95% CI: −0.436, −0.003), and viability (β regression coefficient: −2.66, 95% CI: −4.991, −0.392). No association between urinary BPA concentration and embryo development parameters at IVF/ICSI.	Male age, BMI, current smoking status, alcohol consumption, abstinence period, and urinary creatinine concentration.
Lassen et al. (99)	General population (*n =* 308)	3.25 (0.59–14.89) ng/ml	BPA urine concentration was significantly associated with lower progressive motility (−6.7%; 95% CI: −11.76, −1.63).	Smoking, varicocele, cryptorchidism, genital conditions, and time to motility analysis.
Miao et al. (111)	Factory workers with and without occupational BPA exposure (*n =* 140)	36.23 ± 7.69 μg/grCr in exposed and 1.38 ± 6.89) μg/grCr in non-exposed	Exposed men (*n =* 75) exhibited a lower sperm concentration when compared to unexposed group (*n =* 65): 94.93 ± 58.58 × 10^6^/ml vs. 126.42 ± 82.26 × 10^6^/ml (*p =* 0.03). Higher urinary BPA levels were independently associated with lower global methylation degree of sperm DNA.	Age, education, history of disease, smoking, and alcohol consumption
Dodge et al. (110)	Couples seeking infertility treatments (*n =* 218)	1.6 (0.8–2.8) ng/ml	Lower male BPA concentrations were associated with a greater proportion of high-quality embryos in IVF cycles (RR = 1.92; 95% CI: 1.13, 3.25).	Maternal age, paternal normal weight, maternal normal weight, and maternal smoking.
Goldstone et al. (98)	Male partners of couples who discontinued contraception to become pregnant (*n =* 418)	0.51 (0.46–0.58) μg/grCr^*^	No significant association was found between urinary BPA levels and any standard semen parameter.	Age, abstinence time, alcohol consumption, BMI, smoking, previously fathered pregnancy, center, and ethnicity.
Vitku et al. (103)	Male partners in couples seeking infertility treatment (*n =* 191)	0.075 (0.055–0.100) ng/ml^#^	Seminal BPA but not plasma BPA was negatively associated with sperm count (r^§^ = −0.178; *p =* 0.018), concentration (*r*^§^ = −0.198; *p =* 0.009), and morphology (*r*^§^ = −0.160; *p =* 0.044).	Age, BMI, and abstinence time.
Adoamnei et al. (100)	Healthy young university students (*n =* 215)	1.8 (0.14–11.9) μg/grCr^*^	Urinary BPA concentration was negatively associated with sperm concentration (β regression coefficient = −0.04, 95% CI: −0.07; −0.02) and total sperm count (β regression coefficient = −0.05, 95% CI: −0.08; −0.02).	BMI, smoking, varicocele, abstinence time, and time to motility analysis.
Radwan et al. (104)	Male partners of couples seeking infertility treatment (*n =* 315)	1.64 ± 2.32 μg/grCr	Higher urinary BPA concentration was related to lower sperm motility (*p =* 0.03), increased percentage of immature sperm (*p =* 0.018), and sperm sex chromosome disomy (*p =* 0.01).	Abstinence time, age, smoking, alcohol consumption, and past diseases.
Pollard et al. (106)	Male partners in couples seeking to become pregnant without history of infertility (*n =* 161)	2.5 ng/ml	Higher urinary BPA concentrations were associated with increased percentage of sperm with abnormal tail morphology (*p =* 0.032).	Age, ethnicity, income, smoking, and BMI.

BMI, body mass index; BPA, bisphenol A; CI, confidence intervals; ICSI, intracytoplasmic sperm injection; IVF, in vitro fertilization; RR, rate ratio; VCL, curvilinear velocity.

* values are mean (25th−75th percentiles);

# seminal BPA levels;

§ *r = correlation coefficient of partial correlation; geometric mean*.

Inconclusive results arise from studies on semen quality in the general population. In a study by Lassen et al. (114) on 308 young men enrolled during physical examinations for military service in Denmark, those in the highest quartile of BPA urinary excretion exhibited significantly lower percentages of progressive sperm motility when compared to the lowest quartile group. Adoamnei et al. (115) reported a significant negative association of urinary BPA concentrations with sperm concentration and total sperm count, but not with motility, in 215 healthy young university students. On the contrary, no significant associations were found between urinary BPA concentrations and any standard semen parameter in the Longitudinal Investigation of Fertility and the Environment (LIFE) study, which recruited 418 men from 16 counties in Michigan and Texas (113).

Also inconclusive are the findings on semen quality arising from studies that enrolled men attending fertility clinics. In a study by Meeker et al. (116), where 190 male partners of couple seeking treatment for infertility were dichotomized as either equal/above or below the reference range for total sperm number, sperm concentration, and sperm motility, according to the WHO 1999 criteria (123), urinary BPA concentration was not associated with a significant odd for having semen parameters below the reference levels. Nevertheless, when variables were modeled continuously in multivariable linear regression models, an increase in urinary BPA levels was associated with a slight, albeit just significant, decrease in the percentage of normal sperm morphology (*p* = 0.049), curvilinear velocity at the computer-aided semen analysis (*p* = 0.04) and increased sperm DNA damage (*p* = 0.048) at the comet assay. In a large case–control study by Chen et al. (120), no significant differences were found in urinary BPA concentrations between 877 men with idiopathic infertility and 713 fertile control men. In the same study, crude and multivariable adjusted models did not show significant associations between BPA levels and standard semen parameters (120). In a subsequent study on 149 couples undergoing their first or second *in vitro* fertilization (IVF) or intracytoplasmic sperm injection (ICSI) procedure, an increased urinary BPA concentration in male partners was associated with lower sperm count, sperm concentration, and sperm vitality (117). However, parameters of embryo development (from the fertilization of oocyte to the stage of blastocyst) were not related to the exposure to BPA (117). In another study on 191 Czech men with infertile marriages, seminal BPA, but not plasma BPA, levels were negatively associated with sperm concentration, sperm count, and, to a lesser extent, normal sperm morphology (118). More recently, Radwan et al. (119) reported that urinary concentration of BPA in 315 men with normal sperm concentration according to the WHO 2010 criteria (124) was negatively associated with sperm motility and positively associated with the percentage of sperm sex chromosome disomy. Finally, in a recent report on a preconception cohort of 161 men without known subfertility, higher urinary BPA concentrations were found in the group of men with abnormal sperm tail morphology, whereas no association was found with sperm count, and no information was provided about other semen parameters (121).

In the Study for Future Families (SFF), the only one enrolling men with proven fertility (315 male partners of pregnant women), regression models revealed no relationship between urinary BPA levels and semen parameters (122).

Noteworthy, Li et al. (112) assessed the relationship between urinary BPA levels and semen parameters among 218 factory Chinese workers with or without occupational exposure to BPA. Men with occupational exposure to BPA, who exhibited much higher urinary BPA concentrations, also displayed a significant negative association of BPA with sperm count, viability, and motility. A significant association with lower sperm concentration remained when analysis was restricted to non-occupationally exposed workers. In another study on factory Chinese workers (111), BPA-exposed men (*n* = 75) had significant lower sperm concentration when compared to unexposed group (*n* = 65). Interestingly, authors also found a negative independent association between urinary BPA levels and global methylation degree of sperm DNA, pointing to possible epigenetic consequences of BPA exposure, as already suggested by *in vitro* studies (125, 126).

Actually, two studies assessing more clinically relevant endpoints seem to weaken concerns about the possible adverse impact of BPA exposure on male fertility. In a series from the population-based LIFE study, where Buck-Louis et al. (127) assessed the time to pregnancy in 501 men who were actively trying to conceive, no significant association was found between higher BPA concentrations in the urine and longer duration of pregnancy attempts, after controlling for a number of possible confounders that included partner age. In the cohort of the Environment and Reproductive Health (EARTH) Study, including 218 couples who underwent assisted reproductive technologies (ART) procedures (intrauterine inseminations or IVF), no association was found between paternal urinary BPA concentrations and ART outcomes (128).

### Effects on Sperm Functions: Evidence From *in vitro* Studies

Experimental studies suggest that BPA could extend its biological effects on male fertility beyond the disrupting effect on the regulation of spermatogenesis, by directly affecting sperm functions. In animal models, the *in vitro* treatment with different doses of BPA adversely affected sperm motility in fish (129), bovine (130), mouse (131), and chicken (132) and also impaired sperm fertilizing ability in mouse (131) and chicken (132). Consistent with data from *in vitro* studies on several types of cells (106–108, 133–137), these effects could be mediated by oxidative–apoptotic mechanisms: it has been reported that the exposure to BPA reduces mitochondrial membrane potential (ΔΨm) in chicken spermatozoa (132) and promotes ROS generation in bovine spermatozoa (130); oxidative stress and high DNA fragmentation have been also reported in fish spermatozoa exposed to BPA (129). To date, only two studies have assessed the direct *in vitro* effects of BPA on human spermatozoa. In the first study, which was carried out from our group (138), the exposure of motile spermatozoa to scalar concentrations of BPA (10–800 μM) for 4 h produced a decrease in ΔΨm, starting from 300 μM, which was accompanied by mitochondrial superoxide anion generation, activation of caspase-9 and caspase-3, and motility decrease. As a late consequence of oxidative stress, a 20-h exposure to 300 μM BPA (but not to lower doses) also produced a significant decrease in sperm viability, complete sperm immobilization, and oxidative damage of DNA, as revealed by the generation of the oxidized base adduct 8-hydroxy-2′-deoxyguanosine (138). An inhibitory effect on ΔΨm in human spermatozoa exposed to BPA has been also reported, even at very lower doses, in a subsequent study by Grami et al. (139).

## Discussion

Due to the widespread presence of BPA, environmental exposure to this chemical spares no one: large epidemiological studies revealed that more than 90% of the population in western countries displays detectable BPA in the urine (32–34) and toxicokinetic analyses pointed to dietary and transdermal routes as the primary sources of human exposure. As BPA is qualified as a xenoestrogen endocrine disruptor, growing concern is rising for possible harmful effects on human health, including fertility. Indeed, except for some factory workers with high occupational exposure, measured BPA levels in biological fluids are usually low and the hazards to fertility for the general population remain a matter for debate.

Overall, while preclinical studies have clearly shown that BPA can negatively interfere with the regulation of spermatogenesis, as well as with sperm functions, the claimed clinical adverse effects on male fertility are largely based on the results from conventional semen analysis, that, however, produced controversial evidence (Table 1), being strongly weakened by a number of limitations. Firstly, the cross-sectional design of the studies and the large spontaneous between- and within-subject variability of semen parameters (140) hinder any conclusion about the cause–effect relationships. Although analyses were adjusted for a number of possible confounding factors, it cannot be excluded that other unmeasured confounders have not influenced the examined associations. Other endocrine-disrupting substances are ubiquitous in the environment and may coexist in the human body, leading to possible synergic effects on semen quality with BPA not necessarily playing the major role. Secondly, heterogeneity arises from the inclusion of different study populations with variable degrees of exposure to BPA and, probably, from the variable susceptibility to its effects: in fertile men with low unintentional environmental BPA exposure, any detectable effect on reproductive functions is likely to be small, with uncertain clinical significance. Whether or not low unintentional environmental BPA exposure can worsen the fertility potential in subfertile men would represent a more relevant issue, but it is difficult to be ascertained. On the other hand, when men with or without occupational exposure to BPA were compared, those with occupational exposure, who exhibited much higher urinary BPA concentrations, also displayed a significant negative association of BPA with sperm count (111, 112), viability, and motility (112). Further studies on occupationally exposed workers are warranted.

The best evidence of an adverse effect of BPA on male fertility would be provided by prospective studies on clinically relevant endpoints, including natural or medically assisted pregnancies among men either with different exposure degrees (occupational/environmental) or with different clinical conditions (fertile/subfertile). However, this is a hard challenge.

## Author Contributions

AB and FF contributed conception and design of the study. CC, MT, and AP performed the literature search and wrote sections of the manuscript. SD'A, LL, and GC contributed literature search. AB wrote the first draft of the manuscript. SF, FF, and AB critically revised the manuscript. All authors contributed to manuscript revision, read and approved the submitted version.

## Conflict of Interest

The authors declare that the research was conducted in the absence of any commercial or financial relationships that could be construed as a potential conflict of interest.
